# An acetonitrile solvatomorph of di­chlorido­(1,10-phenanthroline-5,6-dione)platinum(II)

**DOI:** 10.1107/S160053681303256X

**Published:** 2013-12-11

**Authors:** Amanda Hamala, Carissa Fritz, Gulnar Rawji, Vincent Lynch

**Affiliations:** a Department of Chemistry and Biochemistry, 1001 E. University Ave., Georgetown, Texas 78626, USA; b Department of Chemistry and Biochemistry, University of Texas at Austin, Austin, Texas 78712, USA

## Abstract

In the title complex, [PtCl_2_(C_12_H_6_N_2_O_2_)]·CH_3_CN, the Pt^II^ atom lies in a slightly distorted square-planar arrangement defined by an N_2_Cl_2_ donor set. In the packed structure, columns of complex moieties are stacked such that the neighboring units are oriented at 180° and laterally displaced with respect to each other. This prevents any overlap of the phenanthroline rings and thus there is no possibility of any π–π inter­actions between aromatic rings.

## Related literature   

For condensation of the free and complexed phendione ligand with primary amines, see: Dickerson & Summers (1970 ▶); MacDonnell & Bodige (1996 ▶); Moucheron *et al.* (1997 ▶); Westerlund *et al.* (2005 ▶); Williams *et al.* (2012 ▶). For use of the ligand in the construction of multinuclear homo- and heterometallic complexes as well as dendritic polynuclear metal structures, see: Fox *et al.* (1991 ▶); MacDonnell & Bodige (1996 ▶); Paw & Eisenberg (1997 ▶); Calderazzo *et al.* (1999 ▶); Campagna *et al.* (1999 ▶); Calucci *et al.* (2006 ▶). For anti­microbial activity of the free ligand and the title complex, see: Roy *et al.* (2008 ▶). For previous structural studies related to the title complex, see: Granger *et al.* (2005 ▶); Okamura *et al.* (2006 ▶); Roy *et al.* (2008 ▶). For synthesis of Pt(DMSO)_2_Cl_2_, see: Romeo & Scolaro (1998 ▶).
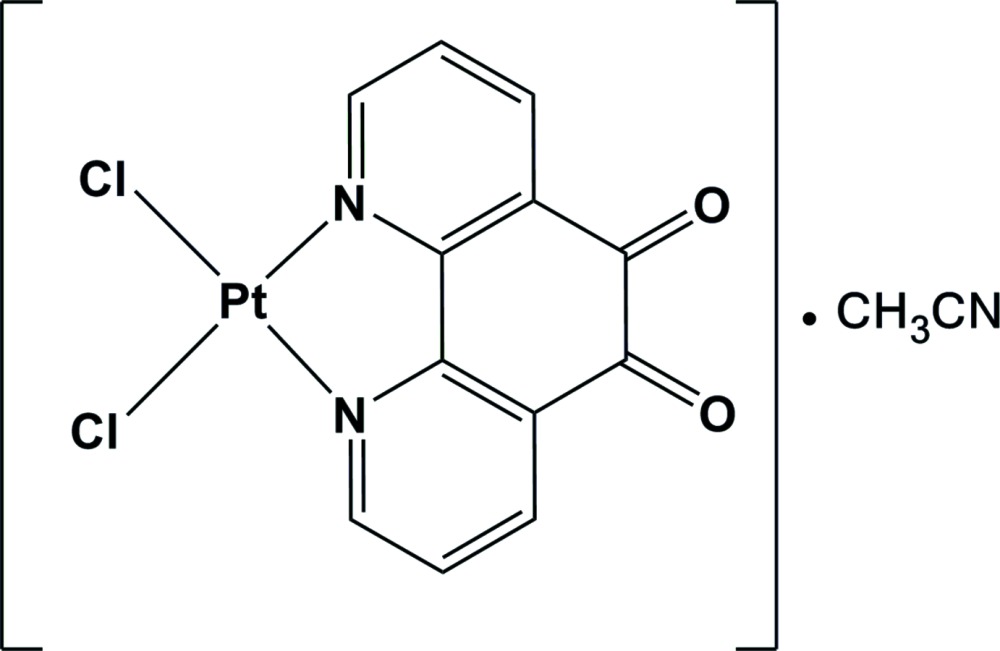


## Experimental   

### 

#### Crystal data  

[PtCl_2_(C_12_H_6_N_2_O_2_)]·C_2_H_3_N
*M* *_r_* = 517.23
Monoclinic, 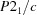

*a* = 6.7285 (2) Å
*b* = 22.6380 (6) Å
*c* = 9.7561 (3) Å
β = 98.0740 (17)°
*V* = 1471.32 (7) Å^3^
*Z* = 4
Mo *K*α radiation
μ = 9.91 mm^−1^
*T* = 298 K
0.40 × 0.06 × 0.05 mm

#### Data collection  

Nonius KappaCCD diffractometer
Absorption correction: gaussian (*XPREP* in *SHELXL*/PC; Sheldrick, 2008 ▶) *T*_min_ = 0.174, *T*_max_ = 0.629
11801 measured reflections
3345 independent reflections
2837 reflections with *I* > 2σ(*I*)
*R*_int_ = 0.081

#### Refinement  

*R*[*F* ^2^ > 2σ(*F* ^2^)] = 0.039
*wR*(*F* ^2^) = 0.094
*S* = 1.16
3345 reflections
201 parameters
H-atom parameters constrained
Δρ_max_ = 1.48 e Å^−3^
Δρ_min_ = −1.30 e Å^−3^

### 

Data collection: *COLLECT* (Nonius, 1998 ▶); cell refinement: *COLLECT*; data reduction: *DENZO* and *SCALEPACK* (Otwinowski & Minor, 1997) ▶; program(s) used to solve structure: *SIR97* (Altomare *et al.*, 1999 ▶); program(s) used to refine structure: *SHELXL2013* (Sheldrick, 2008 ▶); molecular graphics: *XP* in *SHELXTL/PC* (Sheldrick, 2008 ▶); software used to prepare material for publication: *publCIF* (Westrip, 2010 ▶).

## Supplementary Material

Crystal structure: contains datablock(s) I. DOI: 10.1107/S160053681303256X/mw2118sup1.cif

Structure factors: contains datablock(s) I. DOI: 10.1107/S160053681303256X/mw2118Isup2.hkl

Additional supporting information:  crystallographic information; 3D view; checkCIF report

## Figures and Tables

**Fig. 1.**
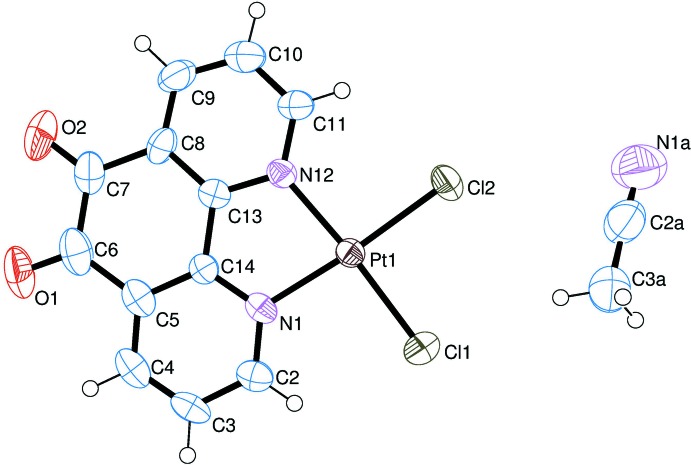
An ORTEP diagram of the title complex showing 50% probability displacement elipsoids.

**Fig. 2.**
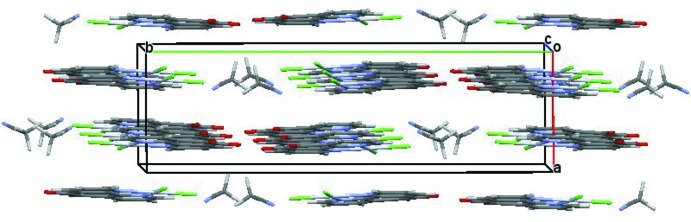
A packing diagram of the title complex viewed down the c axis.

## supplementary crystallographic information

### 1. Comment

1,10-phenanthroline-5,6-dione (phendione), a versatile electroactive ligand with dual functionalities (diquinoid and di­imine), has been widely used in condensation with primary amines either as a free phendione entity (Dickerson & Summers, 1970) or one coordinated to a metal center (MacDonnell & Bodige, 1996; Moucheron *et al.*, 1997; Westerlund *et al.*, 2005; Williams *et al.*, 2012). Because of its dual functionalities, the phendione ligand has been used as a bridging ligand in homo- and heterometallic multinuclear complexes and polymetallic dendritic structures (Fox *et al.*, 1991; MacDonnell & Bodige, 1996; Paw & Eisenberg, 1997; Calderazzo *et al.*, 1999; Campagna *et al.*, 1999; Calucci *et al.*, 2006). The anti­microbial properties of this complex and the free ligand have also been investigated (Roy *et al.*, 2008). In our studies, this ligand and the title complex have been used in the synthesis of metallo­inter­calators of DNA.

Three structural studies of di­chloro­(phendione)platinum(II) have been reported (Granger *et al.*, 2005; Okamura *et al.*, 2006; Roy *et al.*, 2008). In all three studies, the complex was obtained as a DMSO solvate in the Cc space group. Two of these studies appear to be preliminary (Granger *et al.*, 2005; Okamura *et al.*, 2006) and the structural data were not submitted to the Cambridge Structural Database (CSD). The third study is thorough and its deposited data (CCDC 678530) are available for comparison (Roy *et al.*, 2008). The structure reported here is an aceto­nitrile solvate belonging to a different monoclinic space group, *P*2_1_/*c*, resulting in a very different three dimensional packing arrangement compared to that in the reported study.

The title complex is essentially planar with three of the four bond angles about the Pt atom deviating slightly from the idealized square planar geometry. The N12—Pt1—N1 angle is significantly reduced to ~81° due to the constraints of the phendione ligand and two of the remaining bond angles are ~95° (Fig. 1). The unit cell contains four molecules each of the complex and the solvent, aceto­nitrile. Inter­molecular inter­actions are responsible for the observed three dimensional array which may be described as consisting of zigzagging columns of stacked complex moieties, oriented 180° and laterally displaced with respect to each other. The separation between the stacked moieties alternates between 3.360 Å and 3.364 Å but due to the relative orientation of the stacked units, π-π stacking is not possible. The solvent molecules lie between the columns in the space created by the zigzagging (Fig. 2).

The three dimensional array of the title complex is clearly different from that of the reported structure (Roy *et al.*, 2008). A major contributing factor to the different packing arrangements observed in the two studies is the relative orientation of the stacked complex moieties with respect to each other, 180° (current study) versus ~120° (reported (Roy *et al.*, 2008)). The 120° orientation allows a partial overlap of the end rings of the stacked phendione ligands thereby increasing the potential for π-π inter­action. Therefore, the structural study reported here is consistent with a solvate polymorph of [Pt(phendione)Cl_2_] in which aceto­nitrile as the solvent leads to a different packing arrangement than in the DMSO solvate structure.

### 2.1. Synthesis and crystallization

The phendione ligand, K_2_PtCl_4_, and other reagents used in the syntheses were purchased from Sigma-Aldrich. [Pt(DMSO)_2_Cl_2_] was prepared from K_2_PtCl_4_ and DMSO according to the literature (Romeo & Scolaro, 1998). The title complex was synthesized by adding the solid ligand in a 1:1 molar ratio to a stirred aceto­nitrile solution of [Pt(DMSO)_2_Cl_2_] at maintained at 343 K. The pale yellow mixture was kept stirred at this temperature for ~2 hours while replenishing the solvent as necessary. During this time, as the color darkened, orange needles began to precipitate. The mixture was removed from heat and left covered at room temperature for slow crystallization. Good quality light orange needle-shaped crystals suitable for a crystal structure were obtained after ~6 hours. Yield: 40%.

### 2.2. Refinement

The H atoms were placed in idealized positions (aromatic C—H distances of 0.93 Å and methyl C—H distances of 0.96 Å) with displacement parameters *U*_iso_(H)set to 1.2*U*_eq_(C) for aromatic and 1.5*U*_eq_(C) for methyl protons.

### Crystal data

**Table:**

[PtCl_2_(C_12_H_6_N_2_O_2_)]·C_2_H_3_N	*F*(000) = 968
*M**_r_* = 517.23	*D*_x_ = 2.335 Mg m^−^^3^
Monoclinic, *P*2_1_/*c*	Mo *K*α radiation, λ = 0.71073 Å
*a* = 6.7285 (2) Å	Cell parameters from 3185 reflections
*b* = 22.6380 (6) Å	θ = 1.0–27.5°
*c* = 9.7561 (3) Å	µ = 9.91 mm^−^^1^
β = 98.0740 (17)°	*T* = 298 K
*V* = 1471.32 (7) Å^3^	Needle, yellow
*Z* = 4	0.40 × 0.06 × 0.05 mm

### Data collection

**Table:**

Nonius KappaCCD diffractometer	2837 reflections with *I* > 2σ(*I*)
Radiation source: sealed tube	*R*_int_ = 0.081
φ and ω scans	θ_max_ = 27.5°, θ_min_ = 2.3°
Absorption correction: gaussian (*XPREP* in *SHELXL*/PC; Sheldrick, 2008)	*h* = −7→8
*T*_min_ = 0.174, *T*_max_ = 0.629	*k* = −29→26
11801 measured reflections	*l* = −12→9
3345 independent reflections	

### Refinement

**Table:**

Refinement on *F*^2^	Secondary atom site location: difference Fourier map
Least-squares matrix: full	Hydrogen site location: inferred from neighbouring sites
*R*[*F*^2^ > 2σ(*F*^2^)] = 0.039	H-atom parameters constrained
*wR*(*F*^2^) = 0.094	*w* = 1/[σ^2^(*F*_o_^2^) + (0.0216*P*)^2^ + 7.4291*P*] where *P* = (*F*_o_^2^ + 2*F*_c_^2^)/3
*S* = 1.16	(Δ/σ)_max_ = 0.002
3345 reflections	Δρ_max_ = 1.48 e Å^−^^3^
201 parameters	Δρ_min_ = −1.30 e Å^−^^3^
0 restraints	Extinction correction: *SHELXL2013* (Sheldrick, 2008), Fc^*^=kFc[1+0.001xFc^2^λ^3^/sin(2θ)]^-1/4^
Primary atom site location: structure-invariant direct methods	Extinction coefficient: 0.0038 (4)

### Special details

**Table:**

Geometry. All e.s.d.'s (except the e.s.d. in the dihedral angle between two l.s. planes) are estimated using the full covariance matrix. The cell e.s.d.'s are taken into account individually in the estimation of e.s.d.'s in distances, angles and torsion angles; correlations between e.s.d.'s in cell parameters are only used when they are defined by crystal symmetry. An approximate (isotropic) treatment of cell e.s.d.'s is used for estimating e.s.d.'s involving l.s. planes.

### Fractional atomic coordinates and isotropic or equivalent isotropic displacement parameters (Å^2^)

**Table:**

	*x*	*y*	*z*	*U*_iso_*/*U*_eq_	
Pt1	0.22275 (4)	0.538338 (12)	0.35597 (3)	0.03226 (13)	
Cl1	0.2456 (3)	0.63727 (10)	0.4031 (2)	0.0535 (5)	
Cl2	0.1736 (3)	0.55973 (11)	0.12469 (18)	0.0490 (5)	
O1	0.2795 (12)	0.3386 (4)	0.8277 (7)	0.078 (2)	
O2	0.2372 (13)	0.2739 (4)	0.5941 (9)	0.088 (2)	
N1	0.2643 (8)	0.5130 (3)	0.5574 (5)	0.0332 (13)	
C2	0.2906 (10)	0.5475 (4)	0.6672 (8)	0.0413 (18)	
H2A	0.2927	0.5882	0.6548	0.050*	
C3	0.3157 (11)	0.5248 (4)	0.8018 (8)	0.047 (2)	
H3A	0.3346	0.5501	0.8777	0.057*	
C4	0.3120 (12)	0.4655 (4)	0.8204 (8)	0.050 (2)	
H4A	0.3290	0.4496	0.9093	0.060*	
C5	0.2831 (11)	0.4291 (4)	0.7071 (8)	0.0437 (19)	
C6	0.2705 (13)	0.3630 (5)	0.7205 (10)	0.060 (3)	
C7	0.2476 (13)	0.3264 (5)	0.5856 (10)	0.055 (2)	
C8	0.2270 (12)	0.3572 (4)	0.4480 (9)	0.0442 (18)	
C9	0.2042 (13)	0.3274 (4)	0.3234 (9)	0.055 (2)	
H9A	0.2025	0.2864	0.3205	0.066*	
C10	0.1843 (13)	0.3600 (5)	0.2043 (9)	0.056 (2)	
H10A	0.1715	0.3409	0.1192	0.067*	
C11	0.1828 (11)	0.4203 (4)	0.2087 (8)	0.0428 (18)	
H11A	0.1639	0.4414	0.1262	0.051*	
N12	0.2080 (8)	0.4498 (3)	0.3291 (6)	0.0339 (13)	
C13	0.2300 (10)	0.4186 (4)	0.4489 (7)	0.0342 (15)	
C14	0.2592 (11)	0.4543 (3)	0.5756 (7)	0.0335 (16)	
N1A	0.328 (2)	0.6730 (6)	−0.1587 (14)	0.109 (4)	
C2A	0.2973 (18)	0.6945 (6)	−0.0552 (13)	0.076 (3)	
C3A	0.2662 (19)	0.7226 (6)	0.0677 (12)	0.085 (3)	
H3A1	0.3414	0.7588	0.0775	0.127*	
H3A2	0.3099	0.6972	0.1448	0.127*	
H3A3	0.1259	0.7312	0.0650	0.127*	

### Atomic displacement parameters (Å^2^)

**Table:**

	*U*^11^	*U*^22^	*U*^33^	*U*^12^	*U*^13^	*U*^23^
Pt1	0.03263 (17)	0.03434 (19)	0.03048 (17)	−0.00009 (12)	0.00674 (10)	−0.00010 (11)
Cl1	0.0700 (14)	0.0367 (11)	0.0542 (11)	−0.0022 (10)	0.0102 (9)	−0.0047 (9)
Cl2	0.0597 (12)	0.0558 (13)	0.0317 (9)	−0.0014 (10)	0.0069 (8)	0.0063 (8)
O1	0.095 (5)	0.081 (6)	0.060 (4)	0.013 (4)	0.015 (4)	0.039 (4)
O2	0.127 (7)	0.042 (5)	0.094 (6)	0.005 (4)	0.018 (5)	0.027 (4)
N1	0.024 (3)	0.048 (4)	0.029 (3)	−0.003 (3)	0.006 (2)	−0.001 (3)
C2	0.033 (4)	0.051 (5)	0.041 (4)	−0.002 (3)	0.010 (3)	−0.007 (3)
C3	0.041 (4)	0.071 (7)	0.030 (4)	0.003 (4)	0.009 (3)	−0.010 (4)
C4	0.040 (4)	0.076 (7)	0.034 (4)	0.006 (4)	0.006 (3)	0.009 (4)
C5	0.031 (4)	0.062 (6)	0.039 (4)	0.005 (4)	0.008 (3)	0.007 (4)
C6	0.050 (5)	0.074 (7)	0.057 (5)	0.012 (5)	0.009 (4)	0.024 (5)
C7	0.056 (5)	0.050 (6)	0.060 (5)	0.007 (4)	0.017 (4)	0.018 (4)
C8	0.045 (4)	0.030 (4)	0.058 (5)	0.009 (3)	0.009 (3)	0.008 (3)
C9	0.068 (6)	0.036 (5)	0.063 (5)	−0.002 (4)	0.012 (4)	−0.010 (4)
C10	0.061 (5)	0.056 (6)	0.051 (5)	−0.003 (5)	0.010 (4)	−0.015 (4)
C11	0.042 (4)	0.044 (5)	0.042 (4)	−0.005 (4)	0.007 (3)	−0.009 (3)
N12	0.035 (3)	0.037 (4)	0.031 (3)	0.001 (3)	0.007 (2)	−0.001 (2)
C13	0.028 (3)	0.040 (4)	0.035 (3)	0.002 (3)	0.005 (3)	−0.003 (3)
C14	0.033 (3)	0.037 (4)	0.033 (3)	0.000 (3)	0.005 (3)	0.001 (3)
N1A	0.131 (10)	0.094 (10)	0.105 (9)	−0.019 (8)	0.023 (7)	−0.019 (7)
C2A	0.090 (8)	0.056 (7)	0.086 (8)	−0.001 (6)	0.021 (6)	0.003 (6)
C3A	0.106 (9)	0.074 (9)	0.076 (8)	0.006 (7)	0.019 (6)	0.010 (6)

### Geometric parameters (Å, º)

**Table:**

Pt1—N12	2.022 (7)	C7—C8	1.503 (12)
Pt1—N1	2.028 (5)	C8—C9	1.380 (12)
Pt1—Cl2	2.2860 (18)	C8—C13	1.389 (11)
Pt1—Cl1	2.287 (2)	C9—C10	1.367 (13)
O1—C6	1.177 (11)	C9—H9A	0.9300
O2—C7	1.192 (13)	C10—C11	1.365 (13)
N1—C2	1.318 (10)	C10—H10A	0.9300
N1—C14	1.341 (10)	C11—N12	1.341 (9)
C2—C3	1.398 (11)	C11—H11A	0.9300
C2—H2A	0.9300	N12—C13	1.356 (9)
C3—C4	1.356 (13)	C13—C14	1.466 (10)
C3—H3A	0.9300	N1A—C2A	1.166 (16)
C4—C5	1.370 (12)	C2A—C3A	1.399 (17)
C4—H4A	0.9300	C3A—H3A1	0.9600
C5—C14	1.395 (11)	C3A—H3A2	0.9600
C5—C6	1.506 (14)	C3A—H3A3	0.9600
C6—C7	1.545 (15)		
			
N12—Pt1—N1	81.0 (2)	C9—C8—C7	123.0 (8)
N12—Pt1—Cl2	94.83 (17)	C13—C8—C7	117.4 (8)
N1—Pt1—Cl2	175.8 (2)	C10—C9—C8	118.1 (9)
N12—Pt1—Cl1	175.87 (16)	C10—C9—H9A	121.0
N1—Pt1—Cl1	94.9 (2)	C8—C9—H9A	121.0
Cl2—Pt1—Cl1	89.27 (8)	C11—C10—C9	120.8 (8)
C2—N1—C14	118.9 (7)	C11—C10—H10A	119.6
C2—N1—Pt1	127.2 (6)	C9—C10—H10A	119.6
C14—N1—Pt1	113.8 (5)	N12—C11—C10	121.6 (8)
N1—C2—C3	122.0 (9)	N12—C11—H11A	119.2
N1—C2—H2A	119.0	C10—C11—H11A	119.2
C3—C2—H2A	119.0	C11—N12—C13	118.7 (7)
C4—C3—C2	119.2 (8)	C11—N12—Pt1	127.2 (5)
C4—C3—H3A	120.4	C13—N12—Pt1	114.0 (5)
C2—C3—H3A	120.4	N12—C13—C8	121.1 (7)
C3—C4—C5	119.4 (8)	N12—C13—C14	115.0 (7)
C3—C4—H4A	120.3	C8—C13—C14	123.9 (7)
C5—C4—H4A	120.3	N1—C14—C5	121.6 (7)
C4—C5—C14	118.9 (8)	N1—C14—C13	116.1 (6)
C4—C5—C6	122.0 (8)	C5—C14—C13	122.3 (7)
C14—C5—C6	119.1 (8)	N1A—C2A—C3A	177.3 (14)
O1—C6—C5	123.2 (10)	C2A—C3A—H3A1	109.5
O1—C6—C7	119.4 (11)	C2A—C3A—H3A2	109.5
C5—C6—C7	117.4 (7)	H3A1—C3A—H3A2	109.5
O2—C7—C8	121.7 (10)	C2A—C3A—H3A3	109.5
O2—C7—C6	118.4 (9)	H3A1—C3A—H3A3	109.5
C8—C7—C6	119.8 (9)	H3A2—C3A—H3A3	109.5
C9—C8—C13	119.6 (8)		
			
N12—Pt1—N1—C2	−179.6 (6)	C10—C11—N12—C13	−1.8 (11)
Cl1—Pt1—N1—C2	0.9 (6)	C10—C11—N12—Pt1	177.0 (6)
N12—Pt1—N1—C14	−1.0 (5)	N1—Pt1—N12—C11	−178.5 (6)
Cl1—Pt1—N1—C14	179.4 (4)	Cl2—Pt1—N12—C11	1.6 (6)
C14—N1—C2—C3	0.8 (10)	N1—Pt1—N12—C13	0.3 (5)
Pt1—N1—C2—C3	179.3 (5)	Cl2—Pt1—N12—C13	−179.6 (4)
N1—C2—C3—C4	−0.3 (11)	C11—N12—C13—C8	0.1 (10)
C2—C3—C4—C5	−0.3 (12)	Pt1—N12—C13—C8	−178.9 (5)
C3—C4—C5—C14	0.3 (12)	C11—N12—C13—C14	179.3 (6)
C3—C4—C5—C6	−177.9 (7)	Pt1—N12—C13—C14	0.4 (7)
C4—C5—C6—O1	2.0 (13)	C9—C8—C13—N12	1.0 (11)
C14—C5—C6—O1	−176.2 (8)	C7—C8—C13—N12	−178.6 (7)
C4—C5—C6—C7	−177.2 (7)	C9—C8—C13—C14	−178.2 (7)
C14—C5—C6—C7	4.6 (11)	C7—C8—C13—C14	2.2 (11)
O1—C6—C7—O2	0.9 (14)	C2—N1—C14—C5	−0.8 (10)
C5—C6—C7—O2	−179.9 (9)	Pt1—N1—C14—C5	−179.4 (5)
O1—C6—C7—C8	177.3 (8)	C2—N1—C14—C13	−179.8 (6)
C5—C6—C7—C8	−3.5 (12)	Pt1—N1—C14—C13	1.5 (7)
O2—C7—C8—C9	−3.1 (14)	C4—C5—C14—N1	0.2 (11)
C6—C7—C8—C9	−179.4 (8)	C6—C5—C14—N1	178.5 (7)
O2—C7—C8—C13	176.5 (9)	C4—C5—C14—C13	179.2 (7)
C6—C7—C8—C13	0.1 (11)	C6—C5—C14—C13	−2.6 (11)
C13—C8—C9—C10	−0.4 (12)	N12—C13—C14—N1	−1.3 (9)
C7—C8—C9—C10	179.2 (8)	C8—C13—C14—N1	178.0 (7)
C8—C9—C10—C11	−1.3 (13)	N12—C13—C14—C5	179.7 (6)
C9—C10—C11—N12	2.4 (13)	C8—C13—C14—C5	−1.1 (11)
